# Better automation of beamline control at HEPS

**DOI:** 10.1107/S160057752200337X

**Published:** 2022-04-21

**Authors:** Yu Liu, Xue-Wei Dong, Gang Li

**Affiliations:** a Institute of High Energy Physics, Chinese Academy of Sciences, Beijing 100049, People’s Republic of China

**Keywords:** *EPICS*, beamline control, package management, configuration management, software architecture

## Abstract

At the High Energy Photon Source (HEPS; Beijing, People’s Republic of China), where up to 90 beamlines can be provided in the future, minimization of workload for individual beamlines and maximization of knowledge about one beamline that can be applied to other beamlines is essential to minimize the total complexity in beamline control. Presented in this paper are our efforts to achieve these goals by composing relatively simple utilities and mechanisms to automate tasks, while always remembering to keep our automation solutions simple and clear. After an introduction to our choice of basic software in *EPICS*-based beamline control, the issues encountered in introducing package management to *EPICS* modules, as well as solutions to them, are presented; the design and implementation of the packaging system is concisely discussed. After a presentation of our efforts to reduce the need for self-built multi-device *EPICS* ‘input/output controller’ (IOC) applications by providing reusable modular IOC executables, implementation of easily maintainable multi-IOC setups through the separation and minimization of each user’s IOC configurations is given. The ongoing project of comprehensive beamline services at HEPS to further simplify configuration management on multiple scales, ranging from individual beamline devices to all beamlines at HEPS, is introduced.

## Abstract 2

A simple, flexible packaging system for *EPICS* modules and related software is implemented at the High Energy Photon Source (People’s Republic of China), which also produces reusable modular ‘input/output controller’ (IOC) executables that can be composed to replace many multi-device IOC applications. A configuration convention is suggested that helps to implement easily maintainable multi-IOC setups. Also introduced is our ongoing project of comprehensive beamline services to further simplify configuration management.

## 1. Introduction

The High Energy Photon Source (HEPS) (Jiao *et al.*, 2018 ▸) is a fourth-generation synchrotron radiation facility under steady construction in Beijing, People’s Republic of China. Fourteen beamlines will be provided in 2025, when Phase I of HEPS is planned to open up to the public; up to about 90 beamlines can be provided in further phases. The beamline control system at HEPS (Chu *et al.*, 2018 ▸) is mainly responsible for the proper operation of individual beamline devices and the abstraction of the various device-specific control interfaces for these devices as consistent interfaces. Given the large number of beamlines, it is undoubted that, for unnecessary work to be reduced, reusable designs and plans are essentials instead of luxuries. In this paper, we focus on the beamline control system of the facility; nevertheless, we believe that in other parts like the accelerator control system, or even in other large scientific facilities like free-electron laser facilities and spallation neutron sources, the same principles described here are still applicable, and most techniques can be adapted.

Given the background above, the tasks of beamline control can be considered a kind of computer system administration with a focus on diverse device support and large-scale deployment; with this focus, it is easy to note that tasks for HEPS beamline control need to be performed in simple yet reproducible ways. For beamline control at HEPS, we predominantly use Unix-like operating systems, where off-the-shelf utility programs are abundant and it is very easy to compose these utilities to automate tasks. With the utilities, we can express many tasks as command line code, and further abstract common tasks as scripts; as the amount of manual operations decrease, the tasks cost less time and labour, yet the results become more reliable and maintainable – in other words, both efficiency and reproducibility increase.

If ‘control’ is to be seen as an integral part of the infrastructure for ‘automation’, then the practice above can be seen as ‘automation of automation’; in essence, what we do is programming specific to the domain of system administration. When designing and implementing the beamline control system of HEPS, we find that by seriously considering the programming nature of tasks we can often identify the key factors in problems; we may find surprisingly simple solutions to these problems by the composition of relatively simple mechanisms and utilities, like the use of the *flock* utility to implement critical sections (*cf.* Section 3.2). In this paper, by showing some examples for the practice above, we attempt to convey the idea that it is beneficial to regard beamline control as a serious domain of programming.

As is the case with any other kind of programming, while the abstraction of beamline control tasks reduces complexity, it also brings about additional complexity itself. For multiple reasons, *Experimental Physics and Industrial Control System* (*EPICS*) is chosen as the basis for the device control interfaces at HEPS. We find that when performing certain tasks, by deviating from the ‘orthodox’ *EPICS* practice, we are able to make the task much simpler – sometimes by an order of magnitude or even more, in terms of manual labour or lines of code that each party needs to care about (*cf.* Section 3.1); in some cases, we also deviate from the ‘orthodox’ in certain non-*EPICS* parts of beamline control. Therefore, in this paper, often what we discuss is not only ‘automation of automation’ itself but also our choices of strategy when implementing requirements, and more importantly the rationale behind the choices. In general, we prefer composition of simple tools over off-the-shelf but complex workalikes, to avoid the additional complexity in the latter; we prefer free-form development over potential over-design in early phases when unsure whether the abstraction in our mind would be sufficiently beneficial, to reduce time spent on irrelevant details or even anti-features.

## 2. Package management

### 2.1. Choice of basic software

Due to the requirement of large-scale deployment at HEPS, a first issue to consider is the installation of supporting software for the many kinds of devices used in beamline control. The automation of this task belongs to the field of package management, which revolves around the automated installation of software modules and the automatic management of dependencies. There are many package managers, like *DPKG*/*APT* of Debian, *RPM*/*yum* of Red Hat, *Portage* of Gentoo, *etc.*; there are also cross-distribution package managers like *Conda*, *pkgsrc* and *Guix*, and we are aware of efforts, like those by Bertrand & Harrisson (2019 ▸), to use these kinds of package managers for software involved in beamline control. However, we find that in order to properly handle dependencies, we need to duplicate efforts in packaging software already provided by the distribution; otherwise we would need to resort to the official package manager of the distribution for many dependencies. The latter would not only be inconvenient but also degrade the portability of our packages, which is an important reason to use cross-distribution package managers in the first place. Community efforts like *conda-forge* surely help to avoid packaging dependencies by ourselves, but they essentially act as mini-distributions in parallel with the distribution we already use, with significant implications in development and maintenance. Since we are quite satisfied with the attributes of our distribution (see below), we choose not to use cross-distribution package managers at least as of now.

For various reasons, CentOS is currently the primary operating system running on computers in control systems at our institute. For us, while CentOS is not known for minimalism/simplicity or flexibility, it is usable enough for automation work in control systems. More importantly, the strong compatibility between minor releases (like 7.1 and 7.9) of CentOS makes its basic behaviour (without deep customization to the system itself) quite predictable. Factors like the inclusion of all official base packages for a minor release in single 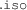 files (like 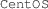 - 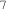 - 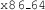 - 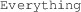 - 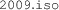 ) and the availability of deployment tools like *Kickstart* (*cf.* Section 3.2) also contribute to easy automated batch deployment. Because of the lifecycle of CentOS releases, we currently base the beamline control system of HEPS on CentOS 7. We are aware of the discontinuation of non-rolling releases of CentOS 8 (Bowen, 2020 ▸) and the resulting uncertainty; depending on future developments of the situation, we will evaluate the migration to a suitable alternative Linux distribution roughly two years prior to the public opening of HEPS Phase I in 2025, before CentOS 7’s end of life in 2024. We estimate that the migration will take roughly two months if we are to use a Red Hat-like distribution, or about six months (with occasional overtime expected) if not. Anyway, for the reasons above, we currently use *RPM*, the official packaging format of CentOS, for beamline control software.

We are aware of the trend in the *EPICS* community to migrate to *EPICS 7*, and the declining activity resulted around *EPICS 3*; considering this background, we plan to use *EPICS 7* whenever applicable, so that we have the best community resources around the version we use. However, due to the relatively immature adoption status of new features (most importantly the PVA protocol) in *EPICS 7* in our testing environment, we deliberately avoid these features in production, and instead use *EPICS 7* like *EPICS 3*. Finally, as is quite common with *EPICS* in beamline control at major synchrotron radiation facilities, we extensively use the *synApps* collection (Mooney, 2010 ▸) of *EPICS* modules for HEPS; this has very profound effects on the packaging of *EPICS* for HEPS, which is discussed in Section 2.2. Here we also note that, although the official documentation of *synApps* does not explicitly state its compatibility with *EPICS 7*, very few build-time or run-time errors have actually occurred in our experience that are traced back to using *EPICS 7*, which proves that *EPICS 7* is mostly backward compatible with *EPICS 3* in terms of functionalities shared between both major versions.

### 2.2. Packaging *EPICS*

*EPICS base* explicitly supports installation to a separate location with few negative effects on downstream code like *synApps* *etc.*, by specifying the 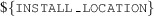 variable which is fairly similar to the 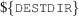 variable supported by many open-source software projects. However, on the current version of the *synApps* homepage, the recommended way to install the software collection is to extract the source code archive into a certain directory (like 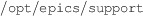 ) in the system, build the source code, and then use the built libraries and executables in place. So there is no separation between building and installation phases with *synApps*, and consequently no clear distinction between source code and files which would be installed separately if 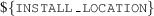 was used. This allows applications to refer to code that is not explicitly to be ‘installed’, *e.g.* *areaDetector* modules referencing files in 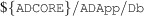 . While perhaps convenient, this also results in implicit dependence on the *synApps* directory layout, which is very unfriendly to packaging.

A first issue we can easily notice is the directory layout. Ideally, there should be preset directories for different types of installed files: executables to a common directory, libraries to another common directory, *etc*. This way, modules just need to refer to these preset directories for necessary files without worrying about where to find each and every module they depend on. There do exist attempts to package *EPICS* modules in this way, with the NSLS-II package repository for *EPICS* and *RTEMS* (BNL, 2018 ▸) being perhaps the most well known example. Nevertheless, as far as we know, none of them provide a satisfactory coverage of modules we are interested in, most importantly *motor* and *areaDetector* modules. From the open-source packaging code we obtain from these projects, we conclude that attempts to use common directories for different modules instead of the *synApps* layout require an inordinate amount of work by packagers, who need to work around references outside 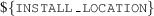 that may occur in too many ways possible to handle in a way representable by simple, clear code. For these reasons, the *synApps* layout is preserved in our packaging system; and not only modules provided by *synApps* are organized in its original way, extra modules, including but not limited to additional motor and area detector modules, are also put into directories similar to the built-in modules they look like.

The second issue is file conflicts. With 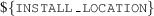 specified, files in the 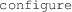 directory are copied into the 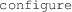 subdirectory of 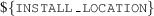 ; no consideration is taken for the coexistence of multiple modules, which may have conflicting 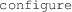 contents. These kind of conflicts also contribute to the difficulty in proper packaging of *EPICS* modules; with the *synApps* directory layout, we do not need to worry about the conflicts between 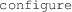 contents of modules, but another kind of file conflict arises. Because our system builds *synApps* and many other *EPICS* modules in place, we cannot directly build them on a computer with themselves already installed, which is highly inconvenient; we used to solve this by running our packaging system in virtual machines, and now we use Docker containers (*cf.* Section 2.3).

The third issue is permissions. The *synApps* directory layout is unrelocatable: the directories, even as a whole, cannot be moved around once it has been built, mainly because of parameters like library search paths and environment variables set to certain absolute paths by the build system of *EPICS*. Because of this, our packaging system builds *synApps* and other *EPICS* modules depending on it in 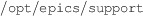 ; to avoid accidentally tampering with system files, the packaging system itself is run as a normal user. However, for the same purpose of avoiding unintended manipulation of packaged files by users, these files should normally be installed into a directory hierarchy owned by the 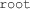 user, and themselves also be owned by 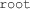 . So in order to build *synApps* and many other modules, we need write access in directory hierarchies owned by 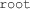 , even though we build packages as a normal user. We resolve this issue by using the *sudo* utility for controlled privilege escalation: we set up password-less *sudo* permission for a ‘builder’ user, and run our packaging system as this user, so it can acquire 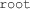 privilege when necessary without manual interaction. The use of 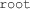 privilege is purposefully minimized: *e.g.* when building *synApps*, we first use it to move a user-owned 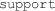 hierarchy, extracted from the source code archive and properly patched, into the 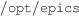 directory; we also use 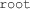 privilege to move the mostly built 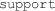 hierarchy into a *staging directory* specified by the packaging tool we use, and then do some necessary processing that can proceed without the privilege. For certain modules (like motor modules that are not yet integrated into *synApps*), some built files may end up in directories (like 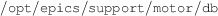 ) where files from other modules can also exist. We handle this kind of situation by temporarily changing the owner of directories with files from multiple modules to the builder user in an early phase, and selectively move the non- 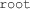 -owned files in these directories into corresponding directories inside the staging directory when the building procedure is mostly done (*cf.* Fig. 2); the selective moving may be regarded as a case where the separation between 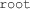 and non- 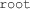 operations actually simplifies the building procedure.

### 2.3. Beyond *EPICS* itself

**Figure 1:**
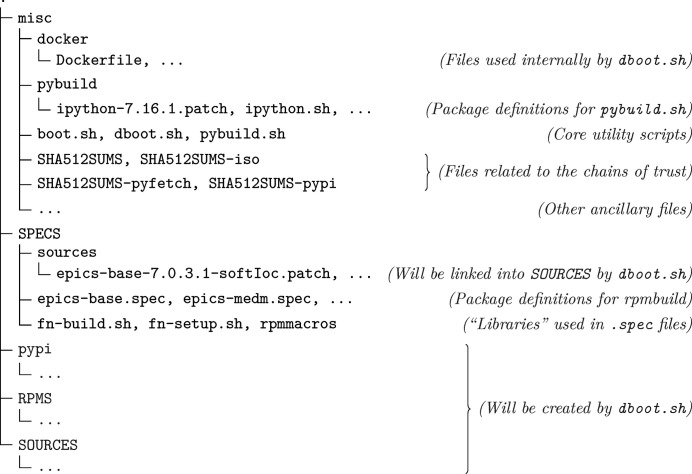
Directory layout of our packaging system.

Considering the limited human resources at our facility, maintainability is a first concern in the design of our packaging system. For this reason, we strive to make the system as ‘small’ as reasonable: we keep the packaging code simple and clear, with proper abstractions that are thin yet flexible enough. A first choice we make is about the organization of packaging code; one approach is to put the code specific to one package alongside the source code archive(s), then generate a *source package*, and finally build the binary package we normally use. This is how Debian-like and Red Hat-like distributions usually work, and is also how the NSLS-II package repository is created; however, a most important drawback of this approach is that the packager may be tempted to apply site-specific modifications directly in source code repositories. In a sense, this disperses packaging code into original upstream code, which creates difficulty in packaging updates and reviewing modifications; we feel this is at least a hidden factor contributing to the difficulty in maintenance of the NSLS-II packages after the leaving of a main developer (Lange, 2021 ▸). Due to the reasons above, we put all our packaging code in a single code repository (Fig. 1 ▸; a fully open-source edition of our packaging system is available at https://github.com/CasperVector/ihep-pkg-ose), and directly build binary packages from there (the source package phase is optional in CentOS 7, and is also not used in our packaging system); this approach is also prevalent in more recent Linux distributions like Alpine and Void.

**Figure 2:**
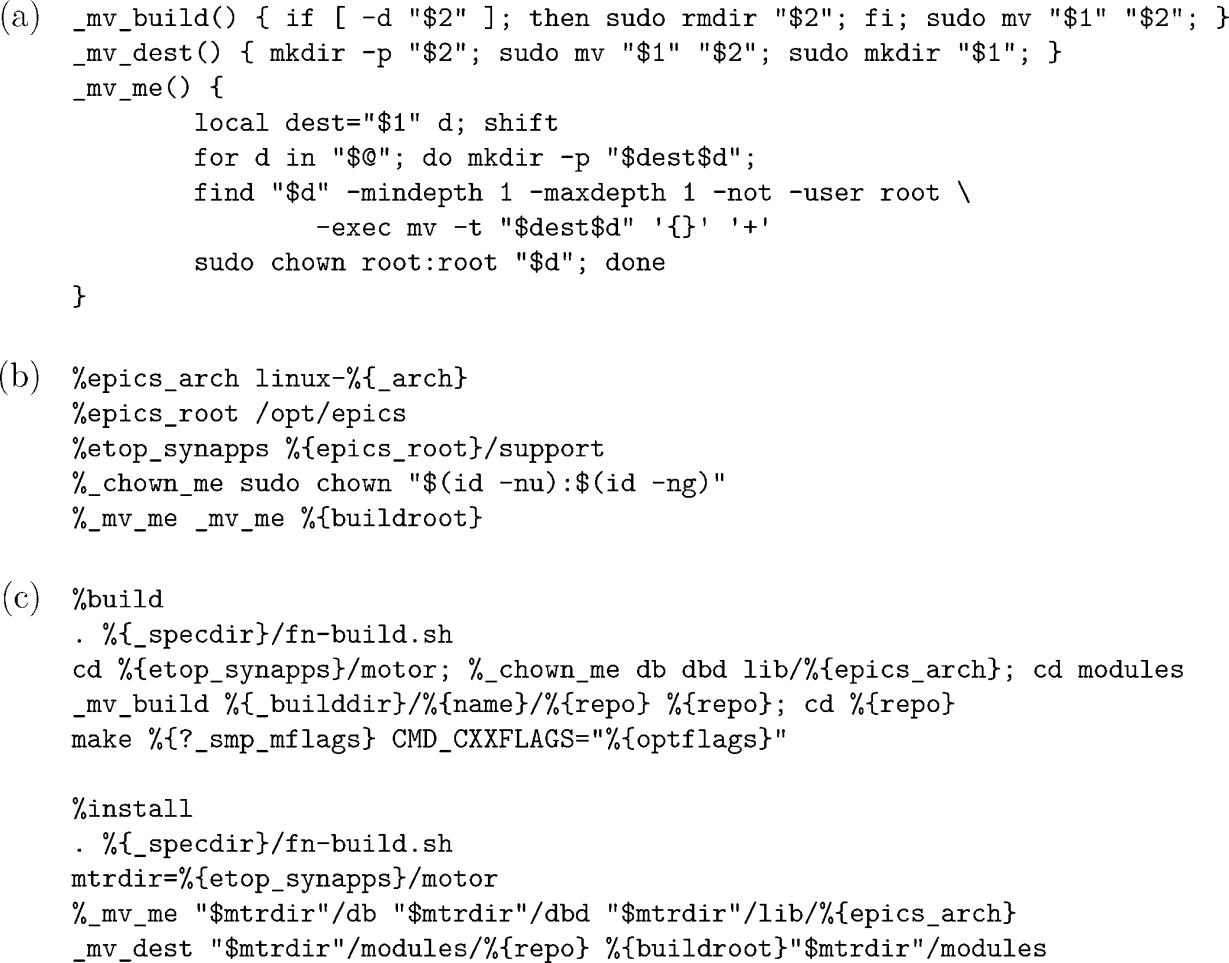
Example snippets from (*a*) 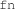 - 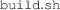 , (*b*) 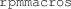 and (*c*) our 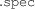 files for extra motor modules (where 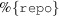 has been set to strings like 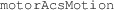 earlier).

**Figure 3:**
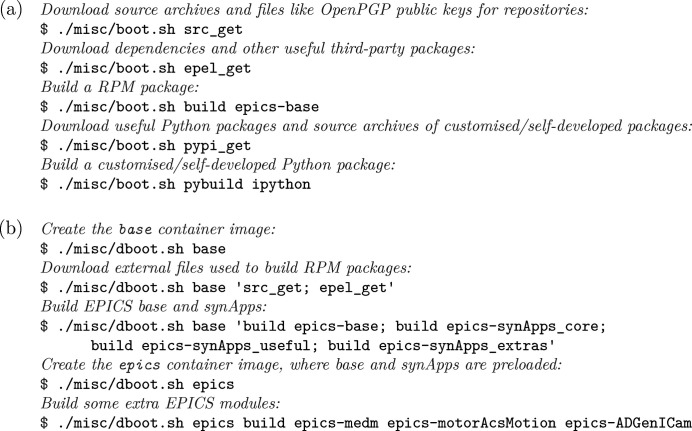
Example usage of (*a*) 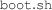 and (*b*) 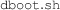 , both expected to be executed from the root directory of the build system.

As has been discussed in Section 2.2 and will be further exemplified in this paper, in order to reduce the intrinsic amount of tasks needed in packaging, we attempt to package *EPICS* and related software modules similar to that intended by their authors with only relatively minor and non-intrusive modifications. To reduce duplication in the actual packaging code, multiple measures have also been taken to abstract common tasks, like how *eclasses* for Gentoo *ebuild* abstract common tasks. Because the majority of packaging code is written in the Shell language, we currently use two library scripts, 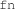 - 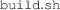 and 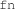 - 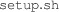 [Fig. 2 ▸(*a*)], to define abstractions for most tasks in the 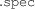 package definition files used in *RPM* packaging; they are not combined into one script because the code in 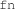 - 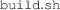 is only used during the building procedure, while the code in 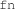 - 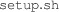 needs to be used when the binary package is installed into the system. Certain common definitions are needed by both 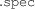 files themselves and the packaging code therein, so we define them in an 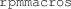 file [Fig. 2 ▸(*b*)] that will be loaded by *rpmbuild*, the official program to build *RPM* packages; also present in 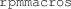 are wrapper macros that pass parameters, which are known to *rpmbuild* but unknown to the library scripts, to functions defined in the latter, so as to further simplify 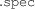 files [Fig. 2 ▸(*c*)]. By the way, although we follow the *synApps* directory layout, we also try to keep a slightly higher level of granularity by packaging basic modules (like *asyn* and *calc*), frequently used modules (like *motor* and *areaDetector*) and other modules as three separate packages [*cf.* Fig. 3 ▸(*b*)]; this not only helps users to slim down systems but also helps packagers to better understand the dependencies.

Apart from running *rpmbuild*, quite a lot of ancillary tasks are also involved in the creation and management of a readily usable repository of binary packages: downloading and managing source archives, downloading build-time or run-time dependencies (*e.g.* *re2c* required by *synApps*) that are not provided by the base CentOS repositories, running *createrepo* to generate necessary metadata for the repository, *etc*. We use a script, 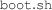 , to abstract these tasks, so that the packager only needs to run simple commands [Fig. 3 ▸(*a*)] to do common tasks. A point worth mentioning is that, instead of downloading dependencies as needed when building a package, we use a locally mounted CentOS 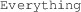 image (*cf.* Section 2.1) to provide all base dependencies, and batch-download all non-base dependencies into the 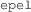 subdirectory of our repository with the 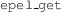 subcommand of 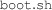 . With the same mechanism, we also download frequently used third-party packages, like *procServ* and Docker, into our repository, so that our users (who are not very skilled Linux users on average) have a one-stop solution for common packages. The source archives can also be batch-downloaded (using the 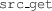 subcommand); the fact that all source materials can be batch-downloaded enables us to pre-download the materials, transfer them to a network-isolated machine with tens of CPU cores, and then perform the building procedure alone on said machine, thus exploiting high-performance machines without regular network access for big tasks.

**Figure 4:**
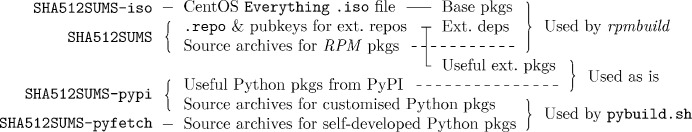
Chains of trust for external files in our packaging system.

**Figure 5:**
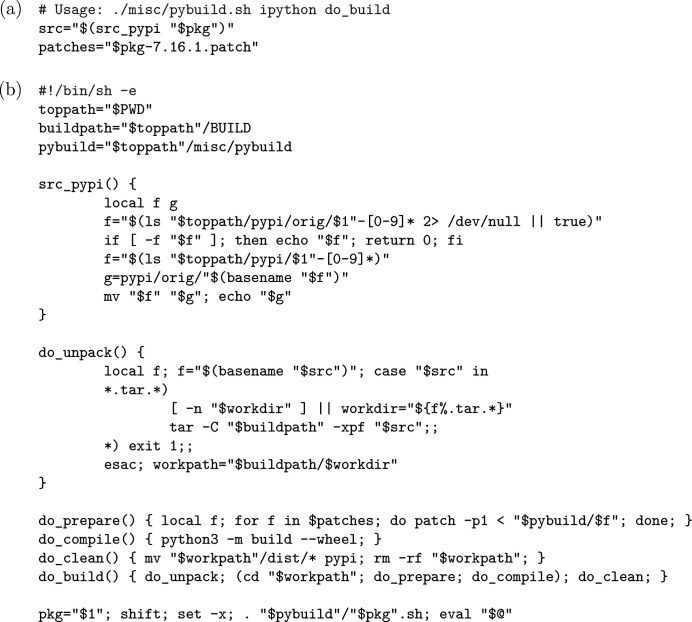
(*a*) 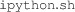 , the definition file of the *ipython* package customized at HEPS; (*b*) a condensed 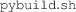 , expected to be executed from the root directory of the build system.

Traditional *RPM* packaging does not involve the integrity check of source archives, which is a common feature in many packaging frameworks, and also a crucial security feature given the recently increasing threats posed by *supply chain attacks*. To compensate for this shortcoming, we maintain a group of checksum files ( 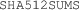 *etc.*) and compare the checksums of downloaded source archives against them at the end of the 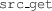 subcommand of 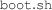 . Actually, for each input outside of our packaging system, we have a *chain of trust* (Fig. 4 ▸) to ensure its integrity: there is 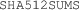 - 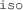 for the CentOS 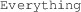 image we use, which ensures every base package contained therein is to an extent trusted; also in 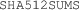 are the checksums for OpenPGP public keys of repositories we download third-party packages (including Docker which is discussed later in this subsection) from, and the signatures of downloaded packages are checked against the keys at the end of 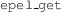 ; the base image which our Docker container images are based upon is also referenced by its checksum to avoid tampering. Another issue loosely related to beamline control is also handled in our packaging system: providing a repository of Python packages [currently mostly related to *Bluesky* (Allan *et al.*, 2019 ▸)] for use in beamline experiments, some of them with customized patches and some of them developed internally at our facility (the adoption of Python-based software at HEPS is still in a fairly early phase, so we do not yet have a formal policy for the management of Python packages other than recommending our repository). We first use the 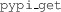 subcommand of 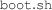 to batch-download most of these Python packages from the Python Package Index (PyPI) and source archives of the several internally developed packages; after this, we can use the 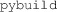 subcommand to build the customized or internally developed packages (the former also downloaded as source archives). The 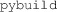 subcommand delegates most of its tasks to a script, 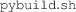 (Fig. 5 ▸), which is essentially a miniature build system for Python packages heavily inspired by Gentoo *ebuild*. We also note that, given the high fluidity of PyPI packages, a ‘hidden’ advantage of our internal Python package repository is the relative stability of systems using the packages therein, assuming our repository is not updated too frequently.

As has been mentioned in Section 2.2, we used to run our packaging system in virtual machines to avoid interference with actually used machines where *EPICS*-related packages have been installed; this also reduced the potential security risks resulting from password-less *sudo* permission of the builder user. Nevertheless, the preparation and usage of virtual machine images for packaging tasks is still fairly complex and involves quite a number of manual steps, which reduces reproducibility and consequently reliability; moreover, file transmission between virtual machines and the host machine is not very convenient to perform in an automated fashion. To further increase reproducibility, we developed the helper script 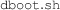 [Fig. 3 ▸(*b*)] to create suitable Docker container images and perform packaging tasks in containers instantiated from these images; built *RPM* and Python packages are respectively put into the 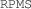 and 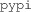 directories (Fig. 1 ▸) automatically, by using the ‘mounting’ feature of Docker. To save time used in installing common build-time dependencies, we distinguish between 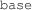 and non- 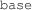 container images: the former used to build basic packages like *EPICS base* and *synApps*, with preparation tasks (like setting up password-less *sudo*) done on image creation time; the latter based on the former but with the basic packages pre-installed (and therefore need to be recreated when these packages are updated). A common practice in software engineering is *continuous integration* (CI), where updates to the code repository trigger rebuild of the changed parts automatically. We currently do not have a fully automatic CI workflow, partially due to the complexity in managing source archives as well as in rebuilding dependencies and container images, and more importantly because the infrastructure needed for CI at HEPS is still under development. However, we also find the current script-assisted rebuilding procedure sufficiently succinct and efficient that it can be regarded as some kind of ‘slightly manual’ CI; fully automatic CI will be completed as the necessary hardware and services are settled down.

## 3. Minimization of configuration

### 3.1. Reusable modular IOC executables

Most of the time in *EPICS*-based beamline control is spent maintaining IOC (‘input/output controllers’) applications which translate various device-specific control protocols into the Channel Access (CA) protocol exposed by *EPICS*. IOC applications are mainly composed of IOC executables (based on underlying support libraries) and data files (most importantly 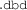 files, 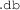 files and 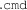 files). From a slightly academic viewpoint, IOC executables are interpreters that interpret 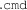 files, which most importantly tell interpreters to load specified 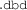 files and 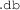 files. Upon closer observation it is not difficult to find that, for IOC applications that handle the same type of devices, the source files of the IOC executables and 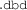 files are mostly unchanged: although the filename of the executable and the 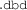 file may change, these factors do not lead to any substantial change in the behaviours of the IOC application. In other words, the exposed interface of an IOC application is mostly affected by the interpreted files excluding 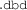 files; we may consider 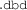 files as an external yet closely coupled part of IOC executables, and other data files as configuration files in general. For many support modules, it is common to provide shared 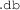 files, and let users compose them into more complex interfaces by using 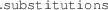 files; we regard these kinds of shared data files as configuration fragments provided upstream that can be composed or customized by the user. From the above, it may be concluded that, for IOC applications dealing with at most one type of device, we may provide reusable IOC executables (including 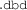 files and other shared data files) that can be fed with different configuration files to act as different application instances. We will show at the end of this subsection that application instances for different devices, seen as modules, can be composed to implement the same requirements done by at least a large fraction of previous multi-device IOC applications; thanks to this change, we can minimize the need for users to build IOC applications by themselves, which greatly reduces the need for systems like *Sumo* (Franksen, 2019 ▸). The applications instances that replace a multi-device application can still be run on the same computer, and be started/stopped as a group; since they would then communicate through the local loopback interface which can only be disrupted in the case of very serious errors, the splitting does not hurt reliability. By properly organizing configuration files for different instances of the same IOC executable (*cf.* Section 3.2), conflicts between different instances can be easily avoided, so we do not need to use mechanisms like Docker to isolate different IOC applications (Derbenev, 2019 ▸), which simplifies deployment and saves both memory and disk space. Another point worth mentioning is that the idea of *reusable modular IOC executables* seems more natural and easier to understand for us than the *require* mechanism (PSI, 2015 ▸), which loads support libraries on demand in 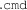 files and requires much more patching in the packaging procedure for every *EPICS* support module involved.

**Figure 6:**
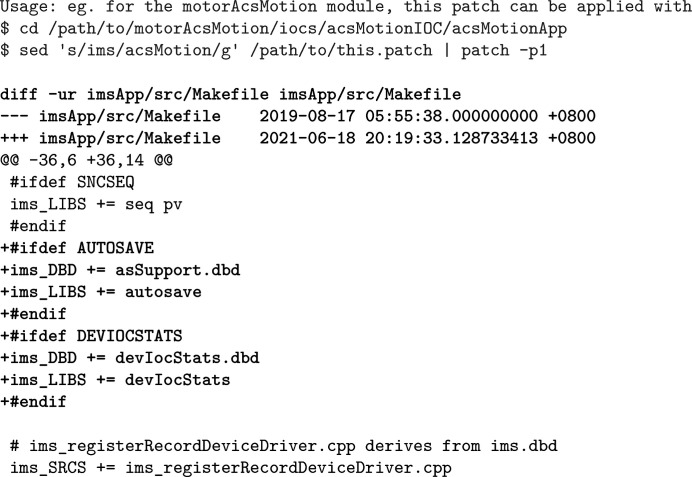
A ‘template’ used to batch-add support for *iocStats* and *autosave* to motor modules.

For reusable modular IOC executables to be provided, they must be built first; for some *EPICS* modules this is done by default, or is disabled but can be easily enabled. For other modules which we want reusable executables of, we need to build them by ourselves; for various purposes, we also add features like *iocStats* (IOC ‘health’ monitoring) and *autosave* (automatic state saving and restore) to most IOC executables. We do these by patching the build system (usually 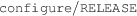 and 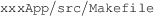 in a module) before actually running it; the centralized management of packaging code (*cf.* Section 2.3) also helps to review patches and compare the patches of modules that are similar in packaging, both of which prove to be very helpful in quality assurance. The build systems of modules that belong to a common type (most importantly motors and area detectors) often only differ in a few highly ‘templated’ locations. This similarity can sometimes be exploited to simplify the patching procedure above: since the patches for these modules would also be templated, we may provide a template (Fig. 6 ▸) and generate the patches on the fly for them based on this template. For this reason, we prefer to make the build systems of internally developed *motor* and *areaDetector* modules similar to their counterparts in *synApps*, instead of using the more ‘standardized’ (and much more bloated) build system generated by traditional tools like 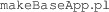 ; to generalize a little, we have the policy for fundamentally similar code in different places that *if they cannot yet be abstracted, at least make them templated*. A situation where we further exploit the similarity between *EPICS* build systems is building generic extra modules: we find that although the build systems of these *EPICS* modules (*e.g.* *Keithley_648x* for Keithley 6485/7 picoammeters and *s7nodave* for Siemens PLCs) do not look immediately similar, they can usually be replaced by a ‘standard’ counterpart with only minimal changes in some ‘template parameters’. For this reason, when packaging *synApps*, we provide a standard 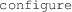 directory and 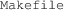 that would appear in 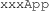 , 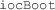 and 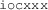 , respectively, at 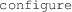 , 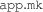 , 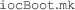 and 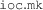 in the 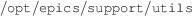 directory, and have successfully used them to replace their module-specific counterparts.

When we discussed reusing IOC executables above, what we meant to reuse was not only those that deal with specific devices but also those that do not directly communicate with devices other than the computers they run on. They may control other devices indirectly by communicating with other applications through the CA protocol, like the *optics* (monochromators and similar utilities) and *sscan* (interlocked action of motors and detectors, functionally like a reduced *Bluesky*) modules; they may also provide PVs (‘process variables’ in *EPICS*) which other applications may read or write through the CA protocol. The behaviours of some applications among them are completely determined by the 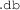 files supplied, to the point that their behaviours remain intact if they are instead fed to the intuitively named *softIoc* executable; this is how we implement these applications, so that we do not even need to write 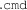 files for them. However, given our requirement of status monitoring mentioned above, we also need to add the *iocStats* feature to our *softIoc* executable [*cf.* Fig. 9(*a*)], which results in a chicken-and-egg situation: *synApps* depends on *EPICS base* which provides *softIoc*, while *softIoc* needs to be added with support for *iocStats* which is from *synApps*. We break this dependency loop by disabling the building of *softIoc* in *EPICS base*, lifting the former’s source code from the latter, using the templates in 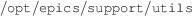 to form the build system for a standalone *softIoc*, and finally building it with *iocStats* support added. Another dependency loop is that the *sscan* IOC executable depends on the *calc* module, which in turn depends on the *sscan* library; we solve it in a similar way – first building libraries in the two modules only, and then building the executables separately. We also note that in order to make references to *EPICS* modules (including executables and libraries) stable across updates, we remove the version tags from the directory names of all *synApps* modules (like the - 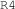 - 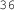 from 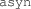 - 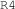 - 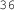 , *cf.* also Fig. 9); this does not prevent the user from querying the version numbers, which can still be found in 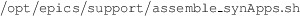 . And to make OPIs (‘operator interfaces’, GUIs for IOCs) easier to find, we create symbolic links to all OPI files under 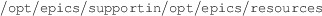 : links to 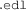 files for *EDM* in the 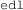 subdirectory, links to 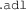 files for *MEDM* in the 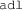 subdirectory, *etc*. Search paths like 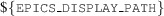 are also set to these resource subdirectories, so that users do not even need to type the directory names for OPI files; in case of ‘missing’ resource files (*e.g.* 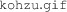 referenced by 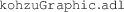 from the *optics* module, expected to be in the same directory), the user can also easily find the real path of OPI files by looking at where the symbolic links point to.

**Figure 7:**
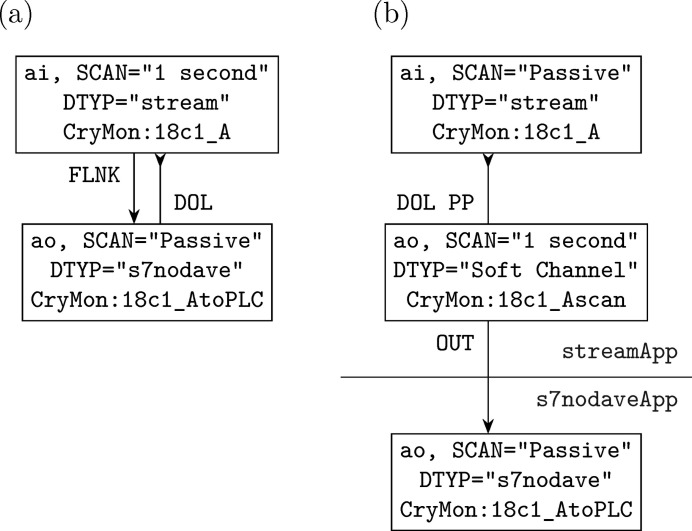
A requirement implemented with (*a*) multi-device and (*b*) single-device IOC applications; for reliability reasons, the latter should be run on the same computer, and be started/stopped as a group.

Interlocked actions between devices are a well known strength of *EPICS*, and we provide full compatibility with self-building of multi-device IOC applications: the libraries and ancillary files are provided as usual, with reusable modular IOC executables just as an added bonus. However, we also find what is done by at least a big fraction of previous multi-device applications can also be done by the composition of their single-device counterparts; the first examples are *optics* and *sscan*, which in our experiments work well whether the devices involved are controlled by the same IOC application or not. From a theoretical perspective, a multi-device application can be split into multiple single-device applications as long as the main modules involved (like *sscan* and the motor/detector modules it is supposed to control) do not actually assume each other were in the same application, or in *EPICS* terms they do not mandate that they communicated through DB links instead of CA links. For some applications, even if this is not true, the link relation between the modules can be refactored to eliminate the demand for DB links; this certainly needs to be analysed on a case-by-case basis, but here we give a relatively simple real-world example that is nevertheless fundamentally similar to many other IOC applications in production, which is why we believe many multi-device applications can actually be split up. For a certain application scenario, we need to poll temperatures from a group of Cryo-con 18C monitor channels, and write them to a group of memory addresses of Siemens PLCs; our previous and current solutions are shown in Fig. 7 ▸. For each pair of temperature channel and PLC address, we use a pair of *EPICS* analogue-input and analogue-output PVs based on *StreamDevice* (*cf.* Section 3.2) and *s7nodave*, respectively. In the previous solution, the *StreamDevice* PV periodically updates (‘scans’) from the temperature channel, and after every update uses a ‘forward link’ to trigger update of the *s7nodave* PV, which pulls the temperature from the *StreamDevice* PV and writes it to the specified memory address. According to the *EPICS* documentation (Kraimer *et al.*, 2018 ▸), a CA forward link only works as expected if it explicitly points to the 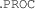 ‘field’ of the destination PV (*e.g.* 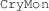 : 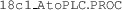 ), so we can eliminate the demand for DB links by batch-adding the 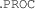 suffix to the forward links. For multiple reasons, we actually use a slightly more complex solution, with ‘soft channel’-based analogue-out PVs (supported by *EPICS base*) as intermediates for temperature values; the ‘soft channel’ PVs are scanned instead of the *StreamDevice* PVs, and the latter are only passively updated (using the ‘process passive’ option) before the *s7nodave* PVs.

### 3.2. Configuration minimization and automated deployment

An issue with introducing package management into *EPICS* is the management of configuration files. The first problem is that, if the user tunes the behaviours of IOC applications by directly changing packaged files, these changes would get lost when the corresponding package is updated; although package managers usually support the declaration of certain packaged files (like all files under 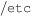 ) as ‘configuration’, which helps to prevent the loss of configuration changes, there are often too many customisable files in *EPICS* to be batch-declared as configuration. The second problem is that since packaged files are typically installed as 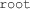 -owned (*cf.* Section 2.2), we often would need to use 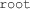 privilege to change them, with implications in security and even convenience: *e.g.* with reusable IOC executables (*cf.* Section 3.1), the same executable may be used by multiple users for different applications, and one user may accidentally overwrite configuration files that actually belong to another user. Both problems can be easily solved if the configurations files by each user are kept inside the user’s own home directory; this is a common practice outside the *EPICS* ecosystem, and is in essence analogous to the separation of packaging code and source archives (*cf.* Section 2.3) in that both distinguish between upstream and downstream (upstream developers *versus * packagers, packagers *versus * users) in order to reduce the amount of code each party needs to maintain. As has been mentioned just now and in Section 2.3, this also helps with software update and migration: a very nice side effect of the separation of configurations is that, if somehow we find a clean way to get rid of the packaging-unfriendly *synApps* directory layout (*cf.* Section 2.2), users will be able to migrate to the new layout relatively easily by batch-changing all path references in configurations files accordingly.

**Figure 8:**
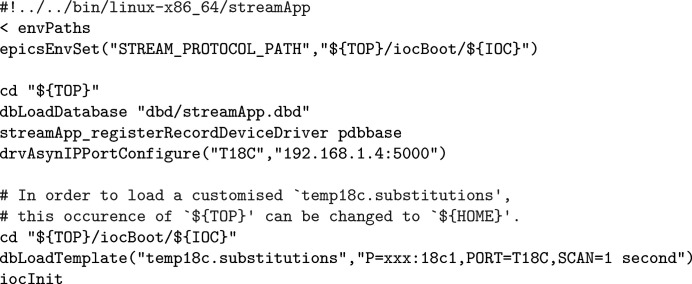
Default 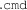 file for Cryo-con 18C temperature monitors.

**Figure 9:**
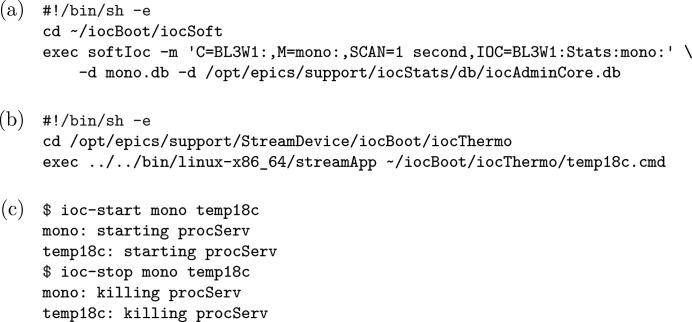
Run scripts in 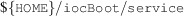 in a production environment for (*a*) a LabView-based monochromator gatewayed to *EPICS* with *CA Lab* ( 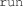 - 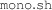 ) and (*b*) some Cryo-con 18C temperature monitors ( 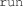 - 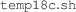 ), with (*c*) example invocations of utilities that start and then stop corresponding applications.

At our facility, we have formed what we call *the ~/iocBoot convention*: putting configurations corresponding to an *EPICS* module (*e.g.* *motorIms* for MDrive motors) into a suitable subdirectory (*e.g.* *iocIms*) of 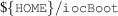 named after the subdirectory of 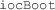 where the configuration files are derived from. This is chosen because 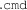 files (Fig. 8 ▸) usually change to the ‘ 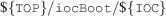 ’ directory before loading their default configurations, so we can easily adapt them by changing the references into ‘ 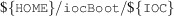 ’ where appropriate. For modules where the 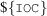 subdirectories are named too generally (*e.g.* 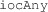 from *optics*), the packager renames them into more distinguishable names (*e.g.* 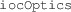 ); for packages where 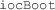 is not provided or the contents are not very good examples, the packager provides self-made examples that can be easily customized. The *StreamDevice* module is widely used in *EPICS* for talking to simple devices in a request/reply fashion, where developers mainly need to provide 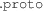 ‘protocol files’ and corresponding 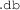 files; we have a dedicated package that collects these files, and groups them into directories like 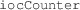 and 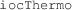 according to the device type [*cf.* Fig. 9 ▸(*b*)]. *softIoc* is a special case where the 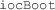 directory is not strictly needed, but in the name of uniformity all 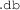 files for *softIoc* are put in 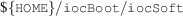 . Furthermore, all 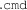 files are patched to store *autosave* state files in 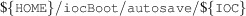 ; nevertheless, to prevent IOC executables from outputting warning messages over and over when the specified directories do not yet exist, the saving and loading of state files are disabled by default. We use the 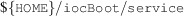 directory to store ‘run scripts’ (Fig. 9 ▸) that when executed run corresponding applications in the foreground; utilities based on *procServ* are also provided to start/stop specified application(s) in the background, and we are developing *procServControl*-based mechanisms to provide GUIs for centralized status management. With all these elements combined, we are able to implement multi-application setups, where the hundreds of IOC applications needed for a beamline (excluding *areaDetector* and other applications that are resource-intensive, as well as VME-based applications) can be accommodated on just a few computers with very modest hardware. We find this much easier to implement and maintain than alternatives, *e.g.* Konrad & Bernal (2019 ▸) which uses much more abstraction based on *Puppet* to compile applications from what is roughly equivalent to ~ 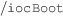 . We note that we are indeed evaluating the use of *Puppet*-like utilities to push changes of centrally managed configurations to beamlines (see below), but that our mechanism will rarely (if ever) need to care about compiling IOC applications.

System administration is closely related to the concept of ‘configuration management’; to us, configuration is the entire procedure of composing hardware and software, whether off-the-shelf or self-developed, into systems that can readily work in production. In the above, we have shown the basic aspects of our efforts to minimize the complexity in configuring software on a single computer for comfortable *EPICS*-based beamline control; in addition to these, we also further reduce the complexity of said configuration by simplifying configuration files using various mechanisms, whether based on *EPICS* (*e.g.* 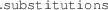 files) or not (mainly code generators, provided the workload saved by them significantly outweighs the workload to maintain them). To also simplify hardware configuration, we have written a software/hardware handbook to give simple yet reproducible instructions that can be followed to configure beamline devices and corresponding software into basic working states. On a larger scale, we are developing a backup system (probably based on *rsync* and related utilities) where the actual configuration of computers (whether running IOCs, OPIs or software like *Bluesky*) are automatically synchronized to a central backup service, which in turn distributes the computer configurations on a beamline to every computer on this beamline, so that the configuration of a computer can be replicated onto a backup computer in case of hardware error. Git-based version control will be applied to the backups, so that we can revisit recent changes in case misconfiguration is suspected; in order to minimize downtime spent on replicating configuration, automation mechanisms will be developed for the replication procedure, and backup computers will be preloaded with basic software after procurement if applicable (at the BDL discussed below). In addition to regular computers, we also plan to extend the backup system to other programmable hardware, like PLCs and network switches, that accept automated and structured configuration input, so that their configuration procedures can also be simplified. A natural step after the backup system is a mechanism to change the configurations centrally and push the changes (also including updates of software packages) to beamlines, perhaps with the help of *Puppet* or similar utilities. We will surely need to solve the technical challenge posed by possible conflicts between changes on both sides, but more importantly we will need to set up administration policies to ensure stability and uniformity of the configurations, while also allowing a suitable level of flexibility for beamline users.

From our definition of ‘configuration’ above, we can see that reducing the amount of configuration for a single beamline device is reducing the amount of device-specific workload, and that reducing the amount of configuration of an entire beamline is reducing the amount of beamline-specific workload. Therefore in order to maximize the scalability of beamline control, we are developing a group of *comprehensive beamline services* (CBS), which covers most aspects in beamline control that are shared between beamlines. A documentation library service will be provided, which gives users access to not only the software/hardware handbook (also including a recommended list of hardware for various uses on a beamline) and the operation manuals of individual beamline devices, but also training materials (which would ‘configure’ new users for various aspects of the facility) and other useful documentation. Centralized network services will be provided, including the backup system and the documentation library mentioned above, our package repositories (*cf.* Section 2.3) and other services like an *EPICS* PV archiver/alarm service and CA gateways to forward information from, for example, the accelerator. For reliability reasons, our control network (where basic control information, represented by CA traffic, is transmitted) will be isolated from the data-transfer network (where experiment data produced by software like *Bluesky* are transmitted); bulk data produced by area detectors are transmitted on the data-transfer network, because of their high throughput and relatively looser requirement on reliability. Beamlines cannot directly communicate with each other, and instead can only communicate with the CBS; transmission of outside information, like NTP information and PVs from the accelerator, is done by gateway services in the CBS. A beamline development laboratory (BDL) will be provided, where beamline devices and major configuration changes (including major software updates) can be tested without interfering with the production environment; the BDL is like a small beamline in terms of networking, of which the CBS is formally a part of.

**Figure 10:**
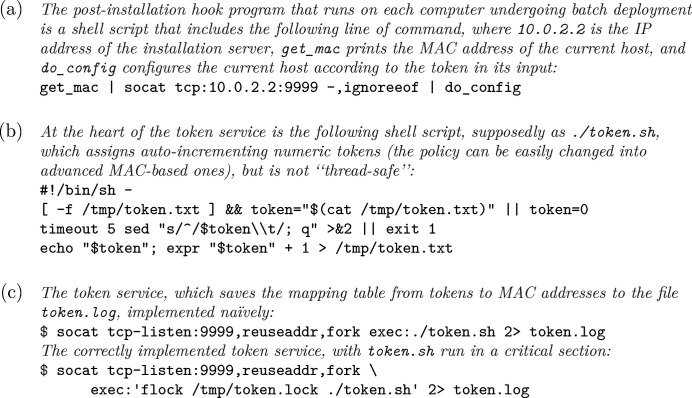
Implementation of a token service.

Another prominent use of the BDL is batch deployment, where basic software is installed on new hardware before the latter is moved to the beamlines or stored as backup hardware. At the end of this paper, we use the batch installation of operating systems onto new computers as an example for how certain requirements in system administration can be implemented in surprisingly simple ways, when we compose simple mechanisms and utilities according to careful analyses of the nature of the problems. Regular installation of operation systems is often performed with CDs/DVDs or USB flash drives, which are exclusive media that cannot be accessed by multiple computers simultaneously; thus for batch deployment, network-based installation (now almost universally based on PXE) is preferred, which is only limited by the network bandwidth of the installation environment and perhaps disk performance of the installation server. In order to facilitate large-scale deployment, network installers of Linux distributions often support some kind of templated unsupervised installation mechanism, like *Kickstart* of CentOS; many of them, including *Kickstart*, also support some kind of mechanism that allows for post-installation execution of specified programs (‘hooks’), so we can automatically preload basic software during unsupervised installation. However, for some requirements, we also need differentiation between computers, *e.g.* installation of systems onto a group of servers for *EPICS* PV archiving with automated configuration of hostnames and related settings. This can be done if we set up a service on the installation server that automatically assigns tokens (which the hostnames will be based on) according to certain rules, and let the hook program obtain the token from the service; using the *socat* utility and a little Shell scripting, this can be done in just a few lines of code (Fig. 10 ▸). The naïve implementation of the service suffers from an obvious race condition when multiple clients ask for tokens at the same time; this can be solved if we execute the service script in a critical section, which can be easily implemented by the *flock* utility (Anvin, 2006 ▸).

## 4. Conclusion

At HEPS, we currently use *RPM* for packaging beamline control software, and deliberately use *EPICS 7* like *EPICS 3*, with most modules based on *synApps*. We preserve the *synApps* directory layout in our packaging system, which however results in potential file conflicts and permission issues; we solve these problems using a builder user with password-less *sudo* permission in Docker containers. The packaging system, including the packaging code and the Docker wrapper, is managed centrally and properly abstracted to minimize complexity. Both self-built packages and some very useful third-party packages are provided in our internal *RPM* repository, and we also provide a similar Python package repository for internal use; all external inputs involved in the creation of both repositories are checked against a chain of trust to avoid tampering. We use reusable modular *EPICS* IOC executables, built with support for *iocStats* and *autosave*, to minimize the need for self-built multi-device applications, and facilitate the use of these executables by providing easy access to resources like OPIs and example configuration files. Full compatibility with multi-device applications is kept, and we also find at least a large fraction of them can be replaced by the composition of their single-device counterparts. We have formed the ~ 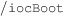 convention to separate each user’s IOC configuration files from the default configurations provided by *RPM* packages, and provide utilities that help to implement maintainable multi-IOC setups for beamlines. Efforts are being made under the umbrella project of comprehensive beamline services to further simplify configuration management on multiple scales: beamline devices and related software on individual computers, all computers and other programmable hardware on an entire beamline, as well as all beamlines at HEPS. Throughout our efforts, we prefer composition of simple tools over off-the-shelf but complex workalikes, and prefer free-form development over potential over-design in early phases, which have helped to boost productivity with our limited resources.
